# Grasping at water: a gap‐oriented approach to bridging shortfalls in freshwater biodiversity conservation

**DOI:** 10.1111/brv.70030

**Published:** 2025-05-06

**Authors:** Charles B. van Rees, Juergen Geist, Angela H. Arthington

**Affiliations:** ^1^ Odum School of Ecology, University of Georgia 140 E Green St Athens GA 30602 USA; ^2^ Institute of Resilient Infrastructure Systems, University of Georgia 597 D.W. Brooks Drive Athens GA 30602 USA; ^3^ River Basin Center, University of Georgia 203 D.W. Brooks Drive Athens GA 30602 USA; ^4^ Aquatic Systems Biology Unit Technical University of Munich Mühlenweg 22 Freising D‐85354 Germany; ^5^ Australian Rivers Institute, Griffith University 170 Kessels Road Nathan Queensland 4111 Australia

**Keywords:** gap analysis, multiple stressors, conservation evidence, adaptive management, nature‐based solutions, environmental flows, wetlands, citizen science

## Abstract

Freshwater biodiversity is the fastest declining part of the global biota, threatened by multiple stressors including habitat loss and fragmentation, climate change, invasive species, water pollution, and abstraction by humans. A multitude of recent agenda‐setting publications have pointed out key objectives and goals for addressing this freshwater biodiversity crisis, but important gaps must be overcome to reach ambitious conservation targets. In this perspective, we complement these high‐level papers in freshwater conservation by highlighting important gaps in knowledge, governance, and implementation. This gap‐oriented approach is designed to facilitate meaningful action by highlighting missing ‘pieces’ in the conservation process, and their connection to existing and emerging solutions in the literature. We derive 13 overarching gaps from a conference session and informal synthesis of recent literature in freshwater biodiversity conservation to catalyse research, advocacy, and action to meet freshwater goals for the post‐2020 Kunming–Montreal Global Biodiversity Framework (GBF). Key gaps include inventory data on global freshwater biodiversity, collating and mobilizing conservation evidence in practice, improving coordination of ecological governance at scale —including within and across catchments—and navigating trade‐offs between economic development, resource consumption, and priorities for freshwater biodiversity. Finally, we apply this gap‐oriented approach to key language describing GBF goals for freshwater biodiversity conservation, and point out existing and emerging solutions which may help address important gaps. Major themes that address multiple gaps include the use of Nature‐based Solutions and Other Effective Area‐based Conservation Measures (OECMs), navigation of water management trade‐offs between human and environmental needs, co‐production of knowledge with Indigenous and local people and other stakeholders, integration of conservation research and action between aquatic and terrestrial ecosystems, and funding and policy mechanisms to facilitate conservation action and support meaningful monitoring of conservation evidence across hydrological scales.

## I. INTRODUCTION

Water is essential for life and central to many of the most daunting sustainability challenges of the 21st century (Rosa *et al*., 2020; van Vliet *et al*., 2021). The conservation of biodiversity in fresh water, the most threatened realm of life on Earth, remains prominent among these, with catastrophic declines across taxa, extensive habitat degradation and impairments in ecosystem services (Barrett *et al*., 2018; He *et al*., 2019; Albert *et al*., 2021). Although decades of excellent scholarship have identified the major threats to freshwater biodiversity as well as the urgent conservation needs of these ecosystems (Dudgeon *et al*., 2006; Strayer & Dudgeon, 2010; Reid *et al*., 2019), insufficient progress has been made in halting the trends of decline and in making freshwater biodiversity conservation more effective (Acreman *et al*., 2020; Erős, Hermoso & Langhans, 2023). There have been multiple calls for simultaneously managing fresh water for the benefit of people and nature (Postel & Richter, 2012; Arthington *et al*., 2018; Anderson *et al*., 2019) and for integrative approaches to freshwater biodiversity conservation considering its value for all life (Geist, 2011; Finlayson, Arthington & Pittock, 2018), and a multitude of recent, rigorous frameworks and priorities point to new pathways for safeguarding freshwater life in a post‐2020 conservation paradigm, including systematic identification of research needs, enabling factors, and actions to overcome barriers to implementation (Tickner *et al*., 2020; Arthington, 2021; Harper *et al*., 2021; van Rees *et al*., 2021; Maasri *et al*., 2022; Barouillet *et al*., 2023).

The Kunming–Montreal Global Biodiversity Framework [Convention on Biological Diversity (CBD), 2022] sets out two targets (targets 2 and 3) for the restoration, effective conservation, and management of 30% of inland waters by 2030 with emphasis on areas of importance for biodiversity and ecosystem services. Accomplishing this goal for inland waters requires a bold new agenda of activities to restore and conserve freshwater biodiversity (Tickner *et al*., 2020; Albert *et al*., 2021). A comprehensive, integrated and more effective approach to sustainable water management and freshwater biodiversity conservation is therefore imperative (Barth, Geist & Cherry, 2023; Cooke *et al*., 2023; Dudgeon & Strayer, 2025). Herein, we refer to freshwater biodiversity conservation in its broadest sense, comprising the full range of actions from relatively static approaches for maintaining genetic diversity, single species or life stages of species, to restoration and protection of functional habitats, ecological processes and ecosystem resilience.

Amongst the many important contributions of this new wave of thinking on freshwater biodiversity conservation, it is worth considering not only goals, actions and prerogatives, but also the gaps between them; the negative spaces through which biodiversity conservation objectives might fall through the metaphorical cracks. Taoist monks used the properties of water as a metaphor for attaining a state of enlightenment, which cannot be achieved by effort alone. Trying actively to grasp and hold water, it runs easily between our fingers, slips away, and is lost. Accommodating the fluidity and mutability of water by cupping the hands instead, it may be contained with little effort or strain. This may be a useful metaphor for approaches to biodiversity conservation (Barmuta, Linke & Turak, 2011) with profound lessons for the struggle for a liveable and just Anthropocene. Perhaps, similar to cupping one's hands to hold water, a different approach is needed in managing a resource as dynamic as water. This includes problems as complex as protecting freshwater biodiversity in a world of declining water security and navigating increasingly frequent conflicts over water use and access. By neglecting the spaces between the many separate ‘fingers’ of academic disciplines, sectors, and cultures seeking to address these complex issues, we risk that freshwater biodiversity, too, will continue to slip away through the gaps.

Complementing recent agenda‐setting scholarship addressing the freshwater biodiversity crisis, we inventory critical gaps that hinder effective and lasting conservation of freshwater biodiversity. In doing so, we highlight unresolved practical issues, disconnected bodies of knowledge, governance barriers, logistical shortfalls, and understudied topics that, if addressed, could increase the integrity and efficacy of global conservation efforts for freshwater ecosystems. We consider gaps as a finer thematic resolution from broader challenges to the field, with the key advantage that they can be more easily matched with existing and emerging solutions. This perspective paper is founded on an informal review of recent literature, presentations from a special session on freshwater biodiversity conservation at the Freshwater Sciences 2023 conference held in Brisbane, Australia, and subsequent synthesis of gaps and emerging solutions.

**Fig. 1:**
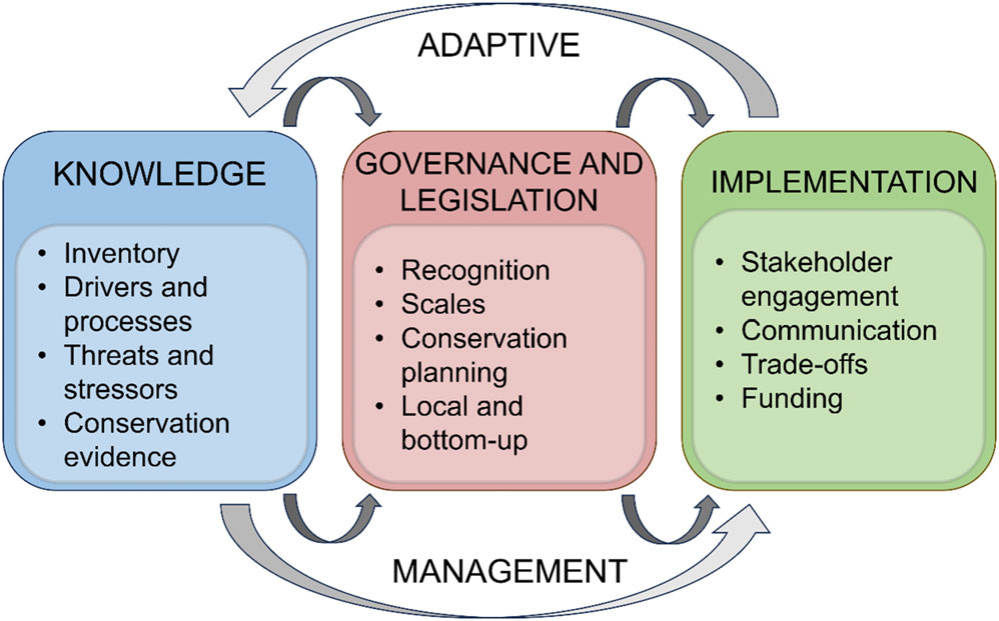
Major gaps across three domains (Knowledge, Governance and Legislation, Implementation) embedded in the adaptive process of freshwater biodiversity conservation, which forms the conceptual basis of this synthetic review.

We organize these gaps into three major domains (*i*) Knowledge (*ii*) Governance and Legislation, and (*iii*) Implementation, based on common themes in influential papers on adaptive management for conservation and natural resources management (Armitage *et al*., 2015; Wyborn *et al*., 2016; Dale *et al*., 2019; Cooke *et al*., 2021, 2023). Each of these domains is part of an idealized cycle of knowledge co‐production, action (i.e. implementation) and adaptive decision‐making (Fig. 1). This approach could ideally be developed into a theory of change which can be optimized based on internal feedback loops. For instance, implementation involving stakeholder engagement and associated communication and consultation strategies can directly contribute to well‐informed conservation evidence, with direct adaptation of government‐based conservation planning. Each gap represents a potential stumbling block or limiting factor preventing desired changes necessary to bend the curve for freshwater biodiversity conservation (*sensu* Tickner *et al*., 2020), and bridging such gaps may facilitate the achievement of change.

## II. KNOWLEDGE GAPS

Two decades ago, Dudgeon *et al*. (2006) called for research to document, appreciate and conserve freshwater biodiversity. Although a vast and expanding literature has since accumulated, important gaps in foundational knowledge and between key disciplines continue to constrain effective conservation and restoration of biodiversity in the freshwater realm (e.g. Geist, 2011; Finlayson *et al*., 2018; Barouillet *et al*., 2023; Table 1).

**Table 1:** Summary and relevant citations for knowledge gaps in freshwater biodiversity conservation.

Gap	Key points	Citations
Biodiversity Inventory	• Biodiversity inventories are lacking for many freshwater ecosystems	Lynch *et al*. (2023a); Jähnig *et al*. (2021)
	• Certain geographical regions such as Africa and South America are under‐represented	Belle *et al*. (2019)
	• Traditional and local ecological knowledge is seldom sought nor integrated in biodiversity inventories	Jackson *et al*. (2022); Souther *et al*. (2023)
Natural drivers and ecological processes	• Integrated river–riparian–floodplain ecosystem models with transferable relationships are lacking	Wymore *et al*. (2023)
	• Prominent gaps in functional geomorphic processes and hydro‐ecological science	Yarnell & Thoms (2022)
	• Poor understanding of terrestrial, freshwater and marine connectivity hinders effective conservation	Thieme *et al*. (2023); Franklin *et al*. (2024)
Stressors	• Failure to address the major causal drivers of biodiversity decline inhibits ecosystem recovery and conservation	Tickner *et al*. (2020); Obura *et al*. (2023)
	• Ecological consequences of small dams and hydropower developments are still poorly understood	Buchanan *et al*. (2022); Zaidel *et al*. (2021); Knott *et al*. (2024)
	• Emerging threats (engineered nanomaterials, microplastics, pharmaceuticals), salinization and artificial light at night) are poorly understood	Cunillera‐Montcusí *et al*. (2022); Hölker *et al*. (2023)
	• Weak understanding of multiple‐stressor effects can lead to inappropriate management practices and unexpected outcomes	Sinnatamby *et al*. (2020); Birk *et al*. (2020)
	• Effects of climate change and multiple stressor interactions on freshwater biodiversity are neglected, weakening opportunities to minimize biodiversity losses	Capon *et al*. (2021); Wild *et al*. (2023); Dudgeon & Strayer (2025)
Conservation evidence	• Governments, practitioners and the public need convincing evidence that conservation policies and practices can effect change	O'Brien *et al*. (2021); Watson *et al*. (2023)
	• Prominent deficiencies in the design, indicators and frequency of protected area evaluations hamper evidence‐based conservation decisions	Acreman *et al*. (2020); Christie *et al*. (2020)
	• Greater evidence for the efficacy and potential contributions of Other Effective Conservation Measures (OECMs) to freshwater biodiversity conservation is needed	Gurney *et al*. (2021); Cook (2023)
	• Evidence is also needed for the freshwater biodiversity conservation benefits of Nature‐based Solutions (NbS), Green Infrastructure, and Natural Infrastructure	van Rees *et al*. (2023a,b); Cuenca‐Cambronero *et al*. (2023)

### (1) The biodiversity inventory gap

Among the most fundamental knowledge gaps in freshwater biodiversity conservation is a comprehensive understanding of the species relying on freshwater habitats and their distributions and phenology. Strayer & Dudgeon (2010) estimated that 10% of all known species spend some or all of their lifetime in fresh water, an estimate that may well increase over time as neglected habitats are explored, and with greater inclusion of groups that are poorly documented in spite of their diverse ecological functions, utility in ecosystem health monitoring, ecosystem services and societal values [e.g. invertebrates, non‐vascular plants (Jähnig *et al*., 2021; Lynch *et al*., 2023a)]. There is also a strong geographical bias in biodiversity inventories with a distinct underrepresentation of Africa, South America, and the tropics in general (e.g. Belle, Stoeckle & Geist, 2019).

Furthermore, several culturally and economically important ecosystem types remain overlooked in research. For example, intermittent rivers and ephemeral streams and episodic arid‐zone floodplain habitats that cease to flow or dry completely at some point in space and time are the world's most widespread type of lotic ecosystem, and their global extent is increasing annually with climatic drying and human water use (Datry *et al*., 2018). However, their biodiversity and ecosystem processes are understudied and largely unprotected in many regions (Steward, Datry & Langhans, 2022).

Groundwater‐dependent ecosystems are another neglected ecosystem type despite holding the largest volume of water of all freshwater biomes and being vital sources of freshwater for human consumption and ecosystem services (Stewart‐Koster *et al*., 2024; Saccò *et al*., 2024). In spite of significant research on hyporheic biota and cave stygofauna, fundamental knowledge of their biodiversity is limited (Hose *et al*., 2022); this is particularly true for aquatic microfauna (Mueller *et al*., 2013). For example, subterranean biodiversity hotspots and unique cave faunas (including the olm salamander *Proteus anguinus*, the world's largest cave amphibian) in the Western Balkans are not captured in the European Biodiversity Strategy for 2030 (Fišer *et al*., 2022).

Although groundwater‐dependent springs are recognized as important reservoirs of relict lifeforms in many countries, they are poorly protected (Cantonati *et al*., 2020). Wetlands in general have been well studied and are prominent in global conservation agendas (e.g., the Ramsar Convention). Yet non‐floodplain palustrine (forest wetlands, peat bogs, marshes and fens), especially small or isolated ones, have been relatively neglected despite their extent, biodiversity value and ecosystem services (Bao *et al*., 2022; Chen *et al*., 2022). Globally, more than half of palustrine wetlands are external to river floodplains and coastal habitats (Lane *et al*., 2023).

The knowledge gaps outlined in these examples are indicative of a much wider deficit of biodiversity inventories and appreciation of ecosystem services to inform freshwater science, restoration, conservation and sustainable use of the world's freshwater ecosystems. Furthermore, most biodiversity inventories exclude or overlook the knowledge of Indigenous people and local communities or include such information only as an afterthought (Souther, Colombo & Lyndon, 2023), neglecting non‐Western perspectives and epistemologies (Anderson *et al*., 2019; Jackson *et al*., 2022).

### (2) The natural drivers and ecological processes gap

Effective ecosystem restoration and biodiversity conservation depend upon a credible foundation of scientific, stakeholder and traditional knowledge, ecological process understanding and a capacity to model, predict and evaluate ecological and societal outcomes from natural processes, human pressures and conservation actions (Cooke *et al*., 2018; Barouillet *et al*., 2023). A rigorous understanding of the drivers of environmental change in freshwater ecosystems, and the ecological processes within them that sustain biodiversity, is a critical knowledge gap inhibiting more effective conservation.

Rivers, riparian systems and floodplains, wetlands, and lakes have been studied for decades yet there remain major gaps in quantifying relationships between the natural drivers and processes that sustain their biodiversity and ecosystem services in space and time (Wymore *et al*., 2023; Finlayson *et al*., 2018). Despite massive efforts to quantify the role of water regimes in driving structure and function in freshwater ecosystems (Poff, Tharme & Arthington, 2017; Yarnell *et al*., 2020), prominent knowledge gaps remain. Examples include interactions between river flow and sediment regimes, erosion and deposition processes and their consequences for channel dynamism, habitat structure and patterns of connectivity to riparian zones and floodplains (Yarnell & Thoms, 2022). Flow‐related connectivity is essential to many species and ecosystem processes in river networks (Pander, Mueller & Geist, 2018), and to wider linkages with other freshwater systems, and the terrestrial and marine realms (Thieme *et al*., 2023). For example, hydrological connectivity governs the export of materials and energy, organism movements across the freshwater–marine interface, and the maintenance of estuarine and within‐channel habitat heterogeneity (Chilton *et al*., 2021). Yet poor understanding and neglect of longitudinal, lateral, vertical and temporal connectivity patterns and meta‐population processes (Cid *et al*., 2022) continue to limit the effectiveness of ecosystem restoration and conservation (Leal *et al*., 2020). Knowledge of drivers and ecological processes gained *via* standard approaches can be supplemented by citizen science and the accumulated knowledge of local and Indigenous people, yet such knowledge is commonly either discounted or sought after in ways that do not respect cultural values and data sovereignty (Jackson *et al*., 2022; McDowell *et al*., 2022).

### (3) The stressors gap

Freshwater ecosystems are disturbed, degraded and destroyed by anthropogenic activities and their byproducts (Reid *et al*., 2019; Tickner *et al*., 2020). Understanding the stressors and drivers of biodiversity decline in freshwater systems is therefore critical for their conservation. The major knowledge gaps around stressors are (*i*) insufficient basic knowledge of novel and emerging threats, and (*ii*) applied knowledge on how different combinations of stressors interact, and how best to manage them. Our failure to recognize and reverse the major drivers of biodiversity decline seriously inhibits ecosystem recovery and conservation (Obura *et al*., 2023).

Numerous small dams (1 m to <7.5 m head height) and culverts have cumulative impacts on stream ecology (Mueller, Pander & Geist, 2011; Geist, 2021), and present social, economic, and safety risks, yet they are poorly managed relative to large dams (Buchanan *et al*., 2022). Depending on the regional context, this is due not only to poor understanding or indifference to ecological damage, but also to lack of incentives, funding and regulatory requirements to address these problems. Irrespective of the reasons, this leads to underestimation of stream fragmentation, habitat loss, effects on stream temperature, water quality, sediment regime and organismal movements (Zaidel *et al*., 2021). Furthermore, the ecological consequences of hydropower developments beyond their effects on economically important fish species are also poorly understood (Geist, 2021) and insufficiently accommodated in policy and water management practice, despite growing knowledge on the topic (Pfeifle, Kennedy & Palmer, 2024). Emerging threats (Reid *et al*., 2019) include new contaminants (engineered nanomaterials, microplastics, pharmaceuticals; Sumpter, Johnson & Runnalls, 2024), acceleration of anthropogenic salinization (Cunillera‐Montcusí *et al*., 2022) and artificial light at night, which can affect diverse behavioural responses and cause physiological harm even at low light levels (Kühne *et al*., 2021; Hölker *et al*., 2023). Interactions among these stressors, and with the multifarious impacts of climate change, warrant far more attention and investigations to mitigate ecological damage (Dudgeon & Strayer, 2025).

Beyond the basic knowledge gaps around novel stressors and their impacts, interactions between multiple stressors and their prioritization for management represent a second form of applied knowledge gap. Most catchments and their waterbodies are exposed to multiple simultaneous threats, each exerting varying types and levels of pressure on species, communities and ecosystems across spatial and temporal scales (Ormerod *et al*., 2010; Craig *et al*., 2017). Urbanization is one of the most pervasive multiple‐stressor syndromes, with serious consequences for freshwater biodiversity (Üblacker *et al*., 2023). Although scientific studies have produced some management guidance to mitigate the impacts of urbanization (e.g. limiting impervious catchment cover to 10% or less; Stanfield & Kilgour, 2006), the interactions of many smaller landscape modifications made by individual property owners, summed over large spatial scales, can amount to large cumulative impacts on watersheds. Multiple co‐occurring threats and interactions can lead to additive, synergistic, and even reversal effects. For example, Wild, Nagel & Geist (2023) point to the underestimated synergistic impacts of increased fine sediment, temperature, and low flow on egg survival of riverine fishes.

Limited understanding of the types, locations, intensity and interactions of environmental stressors, against a background of variation in natural drivers, can lead to inappropriate management practices and unexpected outcomes (Sinnatamby *et al*., 2020). There remain huge gaps in the ‘strategic portfolios of measures’ (Tickner *et al*., 2020, p. 338) that could be transferred and applied to systems with similar combinations of stress. Climate change, itself a complex mix of stressors, already compounds multiple‐stressor syndromes by altering river flow, flooding and drought regimes, while rising temperatures drive higher evaporation rates, water scarcity, changes in water quality and aquatic habitat loss (Sabater *et al*., 2018; Capon, Stewart‐Koster & Bunn, 2021; Wild *et al*., 2023). Disentangling multiple stressors and deciding which particular stresses can be alleviated (and in what order of priority and feasibility) in surface and groundwater systems is a complex process calling for strong guiding principles, interactive stressor–response models and targeted solutions based on available management options (Craig *et al*., 2017; Birk *et al*., 2020).

### (4) The conservation evidence gap

Evidence‐based practice, wherein researchers collate and act upon the results and impacts of conservation interventions, is lacking throughout much of the conservation sector (Sutherland & Wordley, 2017) but will be essential to safeguarding freshwater biodiversity (van Rees *et al*., 2021). Conservation evidence is vital to make best use of available resources, and to motivate practitioners, funders and policymakers with proof of efficacy (Cooke *et al*., 2018, 2023; O'Brien *et al*., 2021; Watson *et al*., 2023). Furthermore, it permits large‐scale syntheses and meta‐analyses, which protect against the influence of poorly designed studies or limited sample sizes that could otherwise lead to costly failures (Reid *et al*., 2022).

Protected areas are an illustrative example of the conservation evidence gap in freshwater systems. Evidence of their effectiveness in conserving freshwater biodiversity is patchy and poorly documented (Hermoso *et al*., 2016; Finlayson *et al*., 2018). A recent evaluation found that only 51% of 75 case studies (from >2,800 publications) contained quantitative evidence of biodiversity benefits in protected areas compared to similar unprotected areas or the same area before designation; the remainder recorded neutral or negative outcomes (Acreman *et al*., 2020). This study and others reveal prominent deficiencies in the design, indicators, and frequency of protected area effectiveness evaluations (Geist & Hawkins, 2016; Christie *et al*., 2020). Evidence gaps around protected areas stem from inadequate or inappropriate control or reference systems, few applications of Before–After–Control–Impact (BACI) designs, and limited recognition of time‐lagged population responses and community outcomes in highly variable systems (Turak *et al*., 2017; van Rees *et al*., 2022). For example, in the case of fishes, it can take decades for species with complex life cycles to show population responses (Pander, Winter & Geist, 2024). Furthermore, limited support for monitoring the outcomes of conservation actions, and lack of reporting and documentation of unsuccessful interventions increases the risk of repeated failures (Geist, 2015).

Conservation evidence is especially important for emerging and high‐priority conservation themes like Other Effective Area‐Based Conservation Measures (OECMs) and Nature‐based Solutions (NbS), both of which may play a large role in meeting the ambitious goals of initiatives like 30 × 30 (CBD, 2022) or half‐Earth (Wilson, 2016), which call for 30% and 50% of Earth's sea and land areas to be protected, respectively. OECMs (e.g. pastoral lands, fisheries‐management areas and Indigenous territories) provide opportunities to strengthen biodiversity conservation alongside formal protected area designations where land and water management objectives align with conservation goals (Finlayson *et al*., 2018; Alves‐Pinto *et al*., 2021; Gurney *et al*., 2021), but empirical evidence of their conservation benefits is presently limited (Cook, 2023). While freshwater NbS like constructed wetlands, levee setbacks, and managed drainage ditches have clear potential to contribute to freshwater biodiversity recovery (reviewed in van Rees *et al*., 2023b), few cases provide robust causal links between actions and meaningful conservation outcomes such as population recovery or enhanced ecosystem function (Cuenca‐Cambronero *et al*., 2023; van Rees *et al*., 2023a). Importantly, implementation of both OECMs and NbS is slow and uncoordinated, limiting the realization of their anticipated impacts (Chausson *et al*., 2020; Cook, 2023). Deficiencies in empirical evaluations of the effectiveness of these and other conservation interventions hamper coordinated efforts, limiting uptake where mainstream applications are needed, and can result in wasted efforts where significant investments are made without evidence of their efficacy (Christie *et al*., 2020; Gurney *et al*., 2021; van Rees *et al*., 2022, 2023b). Furthermore, ambiguity in the technical definition of NbS compounds problems with the slow pace of uptake, leading to uncoordinated and inconsistent implementation (Sowińska‐Świerkosz & Garcia, 2022).

Importantly, the mechanisms driving knowledge creation through conservation evidence (namely a retrospective focus and the use of meta‐analysis or replicated studies) somewhat impede its contributions to this field where interventions are novel (i.e. with few case studies), or where they cover larger spatial scales, giving a less discrete unit of analysis. The former poses a major problem for OECMs and some newer forms of NbS, while the latter may be a broader problem in freshwater systems, where hydrological connectivity and catchment‐level dynamics greatly expand the spatial extent of analysis.

## III. GOVERNANCE AND LEGISLATION GAPS

Effective governance is a primary enabling factor for biodiversity conservation, affecting the equity and efficacy of outcomes and the availability of funding (Armitage, de Loë & Plummer, 2012; Miller, Agrawal & Roberts, 2013; Salerno *et al*., 2021). To guide and precipitate the necessary conservation action, scientific knowledge must be incorporated into governance practices and structures, whether formally as legislation or informally through cultural norms, community values, and expectations. Governance and policy‐related factors are a prominent part of 21st century frameworks for freshwater biodiversity conservation (Tickner *et al*., 2020; van Rees *et al*., 2021), but significant gaps remain in bringing the ambitions of such frameworks into reality (Table 2).

**Table 2:** Summary and relevant citations for governance and legislation gaps in freshwater biodiversity conservation.

Gap	Key points	Citations
Recognition	• Freshwater biota suffer from a ‘public relations crisis’	van Rees (2018); Kalinkat *et al*. (2017)
	• Public awareness and education on freshwater biodiversity is poor	Pascual *et al*. (2023)
	• Freshwater systems are insufficiently recognized in global policy frameworks and are lumped with terrestrial systems in most policies	van Rees *et al*. (2021); Cooke *et al*. (2023); Lynch *et al*. (2023b)
	• The links between Earth systems (especially water), biodiversity and human well‐being are poorly recognized by decision‐makers and the public	Rockström *et al*. (2023)
	• Public under‐appreciation of the scale and rate of biodiversity decline, and difficulty of recovery	Lynch *et al*. (2023b); Obura *et al*. (2023)
Scales	• Societal and ecological variables behave differently across spatial scales, requiring scale‐specific management	Vörösmarty *et al*. (2015); Tickner *et al*. (2020)
	• Water‐management strategies are not coordinated across hydrological scales	Cooke *et al*. (2023); Lynch *et al*. (2023b); Cid *et al*. (2022)
	• Governance and legislation do not accommodate multi‐dimensional connectivity, including longitudinal, lateral, vertical, and temporal connectivity, nor metacommunity and population processes in freshwater networks	Cid *et al*. (2022); Thieme *et al*. (2023)
	• Fragmented power across levels of governance and top‐down regulatory requirements reduce flexibility to deal with uncertainty and change	Geist (2015); Ford *et al*. (2023)
Planning	• Present freshwater biodiversity conservation planning manages only for a stationary future; there is a need for more prospective (future‐looking) planning taking climate non‐stationarity and uncertainty into account	Judd *et al*. (2023); Lynch *et al*. (2023a); Üblacker *et al*. (2023); Harvey & Henshaw (2023)
	• Lack of combined planning of water resource‐oriented and biodiversity‐related objectives	Capon *et al*. (2021)
	• New policy frameworks and governance structures will be needed for biodiversity conservation targets to become part of mainstream infrastructure development and climate change mitigation strategies	Barth *et al*. (2023); Girardin *et al*. (2021); McKay *et al*. (2023); van Rees *et al*. (2023a,b)
Local and bottom‐up	• Disparity between top‐down, target‐setting conservation initiatives and bottom‐up local implementation	Vollmer & Harrison (2021); Vollmer *et al*. (2023)
	• Top‐down conservation and water‐management practices risk inequitable outcomes, with disenfranchisement of Indigenous people and local communities	IPBES (2022)
	• Local buy‐in and governance is a crucial factor in successful conservation measures like Other Effective Area‐Based Conservation Measures (OECMs) and environmental flows (e‐flows)	Arthington *et al*. (2024); Vollmer *et al*. (2023)

### (1) The recognition gap

Before environmental priorities can be sustainably and justly managed, their value, threats, and relation to people must be sufficiently recognized by governance actors, including decision‐makers, policymakers, the public, and local stakeholders. This has been a major problem in freshwater conservation due to the fragmented distribution, limited visibility, and pervasive degradation of freshwater biodiversity, which remains poorly recognized by lawmakers and the public (Moravek *et al*., 2023). At the level of international governance, the unique needs and threats of freshwater habitats and their biota are neglected by their treatment as either part of the terrestrial or aquatic (along with marine systems) realms in important agenda‐setting documents like previous drafts of the Convention on Biological Diversity (van Rees *et al*., 2021; Cooke *et al*., 2023; Lynch *et al*., 2023b); the recognition of inland waters in the new Global Biodiversity Framework may contribute much to alleviating this problem from a top‐down perspective.

At the scale of local or regional governance, scientists highlight a public relations crisis for freshwater biodiversity, which is often less visible to, and recognized by, the public than marine or terrestrial flora and fauna (Kalinkat *et al*., 2017; van Rees, 2018). Specifically, public knowledge of freshwater ecosystems and their biodiversity is poor, and many people remain largely unaware of important fauna and flora and their habitats compared to that in other realms of biodiversity (Pascual *et al*., 2023). Poor public recognition of the severity of the problem and the role of society in perpetuating it results in limited support for critically important conservation initiatives like efforts to reduce resource consumption or consider the downstream consequences of development decisions.

More broadly, both decision‐makers and the public are insufficiently aware of the links between ecosystems, biodiversity, and human well‐being (Rockström *et al*., 2023; Lynch *et al*., 2023b; Obura *et al*., 2023). While urban ‘blue spaces’ supporting freshwater biodiversity are linked to therapeutic effects on people, emotionally and physically (Völker & Kistemann, 2011), and many freshwater species are used directly in traditional and modern healthcare practices (Lynch *et al*., 2023a), public appreciation of these benefits is poor. This lack of awareness makes it difficult to convey the importance of biodiversity conservation and other important measures of environmental sustainability, resulting in legislation reflecting only those aspects of biodiversity which can be exploited to produce market values (Pascual *et al*., 2023). A similar lack of awareness around the concept and negative impacts of habitat fragmentation in freshwater systems was highlighted as a key impediment to effective freshwater biodiversity conservation in a recent review on connectivity (Thieme *et al*., 2023). Assuming good faith in decision‐makers, this lack of recognition or understanding leads to the prevalence of commercial interests in short‐term material gains, typically for a privileged few, over the sustained well‐being of most of our planet's population, especially marginalized Indigenous and local communities. Arguably, the underlying values driving preferences for material wealth over long‐term ecological sustainability stem at least in part from this disconnect, and better public recognition might make good‐faith decisions perpetuating the *status quo* increasingly untenable. Coupled with this issue is an underestimation of the scale and rate of biodiversity decline, and the overwhelming degree of ecological change and degradation being brought on by accelerating exploitation in the Anthropocene (Obura *et al*., 2023). Without a shared understanding of the gravity of the situation, the extent of its progression, and its present and future consequences for human well‐being, it will be difficult for any amount of scientific knowledge and ingenuity in freshwater biodiversity conservation to make their way into governance and eventually into action.

### (2) The scales gap

Decision‐making for water management and biodiversity conservation occurs across multiple jurisdictional, hydrological, and ecological scales, making coordinated governance a major challenge (Tickner *et al*., 2020; van Rees *et al*., 2021). In the simplest sense, freshwater ecosystems like lakes and rivers often cross between or constitute jurisdictional boundaries among states, counties, and provinces, complicating unified governance. Furthermore, the individuals and activities involved with decision‐making at finer (local or project‐level) scales are very different from those at larger (regional, national, organizational) scales, necessitating different skillsets and (inter‐) disciplinary training (Kaufman & Boxshall, 2023). Different disciplinary backgrounds, priorities, and governance processes lead to poor communication and process fragmentation, impeding the coordinated action and functioning necessary for system‐wide restoration and conservation (Bell‐James, 2023; Cooke *et al*., 2023). For example, poor coordination among policymakers, management agencies, scientists, and local communities is cited as a major issue disrupting both conservation interventions and monitoring of freshwater systems in India (De & Dwivedi, 2023).

Differences in problem framing and process among jurisdictional scales can also be counterproductive, as when national‐level policies mandate actions or solutions that are inappropriate for local implementation. For example, in Germany, larger river restoration projects require detailed planning and environmental assessment implementation, sometimes on a level of detail which may need to include the positioning of individual stones in the riverbed (J. Geist, personal observations). As a consequence, the flexibility of restoring ecological processes and their resulting variability can be impaired. Such a scale disconnect between high‐level policies and institutional bodies and local contexts can lead to ineffective implementation and an inability to handle the fundamental uncertainty and potential for unintended consequences inherent to restoration (Ford *et al*., 2023).

The scales gap is also detrimental where environmental legislation affecting only small‐scale decision‐making fails to account for larger‐scale ecological and hydrological processes like runoff, river connectivity and metapopulation dynamics (Cid *et al*., 2022; Thieme *et al*., 2023). While piecemeal, uncoordinated environmental governance may have limited effects on isolated protected areas, the highly networked structure of freshwater metacommunities is negatively affected when neglected by policy (Geist, 2015; Cid *et al*., 2022). For example, failure to account for the impacts of land use on downstream freshwater biodiversity results in a paucity of necessary policy controls to mitigate such hydrologically mediated pressures as nutrient‐laden effluent from agriculture (Pharaoh *et al*., 2024).

### (3) The conservation planning gap

The magnitude of projected hydrological impacts of climate change, burgeoning demand for water and water‐related infrastructure, and coming paradigm shifts in environmental sustainability necessitate new policy and governance structures to support a future‐looking approach to freshwater biodiversity conservation (Darwall *et al*., 2018; Lynch *et al*., 2023a,b; van Rees *et al*., 2023a). Decision‐making processes and supporting legislation are not taking future conditions and uncertainty sufficiently into account (Judd *et al*., 2023). Given the inadequacies of the current, largely static approach to freshwater biodiversity conservation, namely *via* protected areas (Lynch *et al*., 2023b; Miqueleiz, Ariño & Miranda, 2023), future efforts may require proactive approaches that take factors like future climate, land use and water demand into account. While scientific research is producing prospective methods and knowledge, this knowledge is not transferring into governance and legislation, preventing necessary conservation action (Kaufman & Boxshall, 2023).

The prevailing tone of global biodiversity conservation is shifting towards an emphasis on restoration, rewilding, and/or resilience, and balancing societal and ecological goals (Obura *et al*., 2023; van Rees *et al*., 2023a), with ambitious calls for transformative governance and paradigm shifts (IPBES, 2019; Visseren‐Hamakers *et al*., 2021; CBD, 2022). Governance and legislation will be essential to enable and spur such ambitious and systemic change (Bell‐James, 2023). Unless governance structures and legal contexts change to encourage and support conservation planning better for a biodiverse future, uncoordinated and piecemeal actions are unlikely to be sufficient.

While the potential for NbS (and related concepts like Green or Natural Infrastructure) to contribute to freshwater biodiversity conservation goals is increasingly recognized (Cuenca‐Cambronero *et al*., 2023; van Rees *et al*., 2023a,b), the policy and governance capacity to facilitate their development and planning remain a critical need. Major changes in funding allocation, cost–benefit analysis, environmental standards and regulations, and institutional responsibility will be necessary for NbS implementation to provide biodiversity conservation benefits at scale. For example, in the USA, biodiversity *per se* is not easily accounted for as a useful co‐benefit of infrastructure development, leading to a systemic under‐valuation of green infrastructure projects despite their many advantages (McKay *et al*., 2023; van Rees *et al*., 2023b; Chambers *et al*., 2023).

### (4) The local and bottom‐up gap

Prevailing practices in environmental governance and conservation policy result in largely static, technocratic and top‐down approaches to conservation planning and prioritization (Büscher & Fletcher, 2019; Sullivan, Manfredo & Teel, 2022). This creates a substantial governance gap between scientific experts, large‐scale decision‐makers, and local communities. This is an especially large concern for freshwater biodiversity, given the importance of watersheds to local communities and the many scales at which water is managed (see Section III.2). While conservation initiatives may come from academic authorities and higher‐level governing bodies, conservation and sustainability issues often manifest locally and are place‐based, requiring a nuanced and sensitive approach (Vollmer & Harrison, 2021; Vollmer *et al*., 2023). Conservation organizations and higher governance authorities typically do not engage with or understand local politics and culture, undermining the efficacy of conservation solutions and creating risks for perpetuating social inequities and environmental injustice (Vähäkari *et al*., 2020; Ford *et al*., 2023). This is an especially large concern with regard to Indigenous people, whose water‐management practices and ecological knowledge are often neglected and marginalized, but are extremely relevant to place‐based and shared‐use conservation approaches (Parsons & Fisher, 2020; O'Donnell *et al*., 2023).

Local governance and bottom‐up approaches are an essential and mostly missing piece to mainstreaming OECMs and NbS, which require and greatly benefit from local awareness, participation, and engagement (Martin, Fischer & McMorran, 2023; van Rees *et al*., 2023a). Dourado, Rallings & Viers (2023) and Arthington *et al*. (2024) both cite poor integration with formal and informal local governance systems as a major obstacle to wider and more successful environmental flow implementation, noting the importance of making conservation actions relevant and meaningful to local communities. Where the public is unaware of how and why environmental management decisions are being made, conservation projects are less likely to succeed and unintended consequences are a greater risk (Waylen *et al*., 2010; Madani & Shafiee‐Jood, 2020).

Importantly, local‐scale initiatives and priorities will not always be beneficial to biodiversity. Local interests in economic development, water security, or food sovereignty can run counter to the priorities of national or international conservation interests. Such situations will require careful attention to possibilities for win–win scenarios and strategic management of trade‐offs where those are persistent (see Section IV.3).

## IV. IMPLEMENTATION GAPS

Despite the intensity of the scientific discussion and effort on freshwater biodiversity conservation, the translation of knowledge and agendas into conservation action is insufficient to halt the decline of freshwater biodiversity and protect threatened species and ecosystem services. Recently, Barouillet *et al*. (2023) asked why freshwater conservation often gets ‘lost in limnology’ (i.e. why researchers rarely translate scientific knowledge into conservation applications). Twardek *et al*. (2021) suggest an urgent need for mobilizing practitioners to support the emergency recovery plan (Tickner *et al*., 2020) for freshwater biodiversity. Key gaps in implementation of freshwater biodiversity conservation are related to stakeholder involvement, communication, trade‐offs, adaptive management, and funding (Table 3).

**Table 3:** Summary and relevant citations for implementation gaps in freshwater biodiversity conservation.

Gap	Key Points	Citations
Stakeholder engagement	• New conservation paradigms are centred around stakeholder engagement	Anderson *et al*. (2019); Alexander *et al*. (2021)
	• Social equity and environmental justice aspects of stakeholder engagement are insufficiently appreciated	Reid *et al*. (2022); Arthington *et al*. (2024)
	• The knowledge and values of local people, especially marginalized groups [low‐income, Indigenous People and Local Communities (IPLCs), rights holders] must be better integrated into conservation actions	Cooke *et al*. (2023); Thieme *et al*. (2023)
Communication	• Scientific knowledge is not reaching governing bodies, decision‐makers and action agencies	Reid *et al*. (2022); Twardek *et al*. (2021); Harper *et al*. (2021); Judd *et al*. (2023); Kaufman & Boxshall (2023)
	• Language barriers between disciplines and countries impede coordination and knowledge exchange	Amano *et al*. (2023); O'Reilly & Starrs (2023)
	• Differences in discipline, training, value and worldviews require bridging and reconciliation for cooperation	Miranda (2023); Hein *et al*. (2023); Kuiper (2023); Kramer *et al*. (2023)
Trade‐offs	• Lack of methods for navigating trade‐offs between human and environmental water needs	van Rees & Reed (2015); van Rees *et al*. (2019)
	• Available methods are seldom applied to assess conflicts and resolutions	Arthington *et al*. (2024); Widén *et al*. (2022); Dalcin *et al*. (2023); Poff *et al*. (2016)
	• Need to acknowledge inevitable trade‐offs between climate, biodiversity, and human well‐being priorities	Opperman *et al*. (2023); Keskinen *et al*. (2021); Baldwin‐Cantello *et al*. (2023)
Adaptive management	• Lack of evidence hampers adaptive management practices, including technical progress and disciplinary learning	Maasri *et al*. (2022); van Rees *et al*. (2022); Cid *et al*. (2022); Acreman *et al*. (2020); Adams *et al*. (2015); Geist (2015)
Funding	• Funding is insufficient for supporting necessary action	Cooke *et al*. (2023); Arthington *et al*. (2024)
	• Funding is typically too short‐term, and lacking adequate spatial scale	Transforming Evidence Funders Network (TEFN); van Rees *et al*. (2023b)
	• Differential appeal and awareness of issues leads to disproportionate funding for certain systems	Wineland *et al*. (2021)
	• There is a need to combat investments that harm biodiversity	Zu Ermgassen *et al*. (2022)

### (1) The stakeholder engagement gap

Conservation implementation increasingly involves the direct engagement and involvement of stakeholders, ideally throughout the process of goal‐setting, planning, action, and monitoring and evaluation (i.e. co‐production). Despite the existence of stakeholder engagement models (Arthington *et al*., 2024) and practical examples (Barouillet *et al*., 2023; Nagel *et al*., 2025), co‐production remains more an ideal than a common practice in implementation.

Given the ubiquitous importance of freshwater resources for human needs (e.g. food production, waste disposal and sanitation, urban and industrial supply), stakeholder involvement in freshwater management issues is especially critical (Geist, 2015; Geist & Hawkins, 2016), and has been highlighted by many recent agenda‐setting publications in freshwater conservation (Harper *et al*., 2021; van Rees *et al*., 2021; Dourado *et al*., 2023; Vollmer *et al*., 2023). In particular, it is a necessary step for increasing the social equity of conservation decisions and avoiding environmental injustices, and a key component in managing freshwater environments as social‐ecological systems (Cooke *et al*., 2021; Stewart‐Koster *et al*., 2024). Stakeholder co‐production greatly increases project acceptance by the public (Serra‐Llobet *et al*., 2022) and can accordingly facilitate social sustainability of ongoing efforts. Widespread stakeholder engagement for conservation action, especially with regard to water and its diversity of economic and spiritual values to people must respectfully and ethically engage with historically marginalized groups, Indigenous and local people, and other rights holders (Schick *et al*., 2018; Reid *et al*., 2022; Arthington *et al*., 2024). Such efforts must be coordinated across projects, institutions and scales to encourage multiple forms of participation (Chaplin‐Kramer *et al*., 2022).

### (2) The communication gap

Communication gaps are common between the scientific community and decision‐makers as well as action agencies responsible for implementation (Bickford *et al*., 2012). Although conservation priorities and ecological knowledge develop quickly in the peer‐reviewed literature, this knowledge does not easily reach decision‐makers, who may be unable to or uninterested in accessing the peer‐reviewed scientific literature, and intermediate media for communicating key points are limited. More efficient channels for communicating key concepts and priorities for actions to decision‐makers and the public are critically important. For example, user‐friendly outputs and social media platforms can build understanding and knowledge exchange with participant stakeholders and broader communities (Djenontin & Meadow, 2018). A well‐structured engagement and communication strategy can help convey important messages, exchange insights, and help shift the mindsets of a wide range of stakeholders, community groups and decision makers towards biodiversity protection (Arthington *et al*., 2024). Such strategies will benefit from psychology‐informed methods like focusing on values and lived experience and seeking to influence cultural norms, which have large impacts on public and policymaker behaviour (Toomey, 2023).

Different disciplinary languages and terminology influenced by different training backgrounds, worldviews and mind‐sets also require bridging by flexible forms of communication, for example cross‐sectoral meetings or colloquia. Most recently, communication from the public back to the scientific community has received increasing attention, for example leveraging community science (also known as citizen science) as a way to build relationships (e.g. O'Reilly & Starrs, 2023). Such broader participation in conservation science can be very useful to engage the wider public yet can also become problematic when people who want to be part of practical solutions are not properly guided, and even may in some cases do harm (for example, disturbance of endangered species during field observations, or disempowerment of participants; Walker, Smigaj & Tani, 2020). Furthermore, since conservation action typically relies on inter‐institutional workflows, coordination and communication among actors with similar directives and goals is essential to avoid wasted effort and align efforts for greater efficacy (van Rees *et al*., 2021). For example, in the USA, civil engineering and environmental management interests are increasingly aligned around ecosystem restoration for NbS development, but a shared vocabulary and more frequent professional interaction and knowledge exchange are needed to support effective project delivery teams (McKay *et al*., 2023; van Rees *et al*., 2023a).

Language barriers are an underappreciated hindrance to communication among disciplines, sectors, and geographic regions working in support of conservation science. Non‐English speakers are at a distinct disadvantage both in accessing the scientific literature and in sharing important findings *via* peer‐reviewed publication (Amano & Sutherland, 2013; Amano *et al*., 2023). This results in missed opportunities and blind spots in the ecological and conservation literature, reducing its potential for meaningful action. Recent developments in artificial intelligence, including large language models, may help address this bias (Berger‐Tal *et al*., 2024).

### (3) The trade‐offs gap

Freshwater resources management requires accommodating multiple simultaneous demands for freshwater (e.g. water security, energy and food production, transportation, biodiversity conservation). Given projections of rising water demand and increasing scarcity, trade‐offs among water uses for societal and environmental goals are a present concern that is likely to grow in the future (van Rees *et al*., 2021; Vollmer & Harrison, 2021; Baldwin‐Cantello *et al*., 2023; Judd *et al*., 2023). Trade‐offs arise where freshwater biodiversity conservation goals involve curtailing water consumption for societal needs, or where water management for socioeconomic ends directly contributes to ecological degradation (Maasri *et al*., 2022; Judd *et al*., 2023). Ideally, judicious management of trade‐offs can highlight opportunities for ‘win–win’ or mutual gains solutions where societal and conservation goals are met simultaneously, although this may not be realistic in all cases (van Rees & Reed, 2015; van Rees *et al*., 2019). Indeed, such trade‐offs among competing water uses and their public perception, especially in water‐scarce regions, are considered one of the foremost barriers to the implementation of environmental flows (Wineland *et al*., 2021, 2022; Arthington *et al*., 2024), and anticipated hydrological changes in coming decades portend an increase in the frequency and urgency of such situations as societal water security declines (Vanham *et al*., 2021; Erős *et al*., 2023). Accordingly, the anticipation, understanding, and management of trade‐offs between human and environmental water needs is a major priority for the next generation of approaches to freshwater biodiversity conservation (van Rees *et al*., 2021; Widén *et al*., 2022; Opperman *et al*., 2023).

Water allocation among competing demands is a contentious subject in water scarce and arid regions, although they are poorly studied with respect to freshwater biodiversity (Graham *et al*., 2024). Greater rates of imperilment for freshwater ecosystems in such climates suggests that low water security may compromise the allocation of water for environmental needs (Feio *et al*., 2023; van Rees, 2023). Consumptive water uses like river withdrawals for agricultural irrigation in water‐scarce areas are an intuitive manifestation of water trade‐offs, where water allocation to support conservation goals may restrict water use for food production and human livelihoods (Vanham *et al*., 2021; Wineland *et al*., 2022). For example, in the Mujib River in Jordan, extensive groundwater abstraction for upland agriculture compromises baseflow, likely impairing macroinvertebrate communities and contributing to reduced flooding and habitat homogenization (Graham *et al*., 2024). With increasing droughts due to climate change and demands for water to maintain food security under future population growth these competing water demands are likely to increase. Minimizing such trade‐offs and seeking win–win solutions is a critical priority for implementation and governance (Erős *et al*., 2023).

Surprisingly, available methods to assess conflicts and seek trade‐off resolutions are seldom applied in conservation science, despite the existence of a robust literature on water resource conflict management (e.g. Biswas, 2011; Hileman, Hicks & Jones, 2016; Thieme *et al*., 2021) and past efforts to bridge the gap between disciplines like water resources management, environmental flows and conservation biology (van Rees & Reed, 2015; van Rees *et al*., 2019). As with the communication gap between academics and decision‐makers (see Section IV.2), additional communication shortfalls between academic fields like sustainable development, water resources management, and limnology may be partly responsible for these apparently useful frameworks going largely unnoticed in biodiversity conservation. Thus, despite the obvious importance of managing and quantifying trade‐offs in freshwater biodiversity conservation (e.g. Poff *et al*., 2016; Harper *et al*., 2021; Thieme *et al*., 2023; Arthington *et al*., 2024), case studies applying these tools are limited, leaving a prominent gap for achievement of the goals envisioned in the Kunming–Montreal Global Biodiversity Framework. Bridging this gap and better recognizing and managing environmental water trade‐offs may be a key enabling factor for mainstreaming NbS and other synergistic water management solutions (van Rees *et al*., 2023a; Vollmer *et al*., 2023).

### (4) The adaptive management gap

Conservation actions for freshwater biodiversity must necessarily be agile and flexible to cope with the complex dynamics of freshwater social‐ecological systems. Notably, while monitoring is the foundation for adaptive management, funding and support for post‐project monitoring remains rare in conservation (see Section IV.5). Among projects with monitoring, only a small fraction includes a BACI design or comparison to suitable control or reference sites (Geist, 2015; Acreman *et al*., 2020; see Section II.4). The absence of long‐term post‐implementation monitoring and coherent data collection over time facilitating meta‐analyses are also substantial challenges (Maasri *et al*., 2022).

The lack of monitoring is an especially major implementation gap for emerging approaches like NbS, where robust monitoring data are scarce and critical to validate conservation evidence approaches (van Rees *et al*., 2022). Another challenge comes with monitoring at mismatched spatial scales, or without proper consideration of societal variables and their scales of interaction with ecological processes (Cid *et al*., 2022). Whilst responses of some species groups and life stages can be rapid in freshwater systems, effects on the entire life cycles of long‐lived species such as fishes may take decades or longer (e.g. Pander, Mueller & Geist, 2015; Pander, Winter & Geist, 2024). Consequently, time‐lag effects in population and community responses must be considered in post‐implementation monitoring (Acreman *et al*., 2020; van Rees *et al*., 2022). Existing policy frameworks may also limit the adaptiveness and agility of conservation project implementation. Pre‐approval of the plans by the responsible agencies is typically mandatory, locking action agencies into planned procedures and project designs. These approval processes do not allow adjustments based on actual field observations during the conservation or restoration action, limiting improved designs or management strategies revealed by new information (Geist, 2015; see Section III.2). Without such freedom to adapt, long‐term projects will have limited efficacy with respect to the non‐stationary futures forecast under global climate change (Capon *et al*., 2021).

### (5) The funding gap

In most cases, mandating and implementing necessary conservation action requires dedicated and consistent funding for staff and equipment over long periods of time. For instance, a recent survey on conservation efforts related to captive breeding of endangered freshwater mussels across Europe identified limited funding, and especially limited long‐term funding, as one of the major challenges for effective conservation of this highly endangered and long‐lived group (Geist *et al*., 2023). In this case, funding is necessary not only to sustain and monitor habitat restoration and captive breeding efforts for sufficiently long as to allow recruitment in the population (which can be on the order of 10–15 years in some species), but also to evaluate the efficacy of captive breeding programs (Rytwinski *et al*., 2021). In parallel, strategies for financing large‐scale conservation efforts, especially in the 21st century context of restorations and NbS, are also critically needed (van Rees *et al*., 2023a).

Available funding is typically directed towards the most charismatic flagship species within freshwater systems, such as salmonids (Geist, 2011), whereas less‐popular keystone species of great functional importance can be left out. From the standpoint of decision‐makers, highly visible interventions that generate positive media attention are typically more popular than actual process‐based conservation measures such as restoration of nature‐like flow regimes with more complex and less obvious or prolonged outcomes (Arthington *et al*., 2024). This bias similarly neglects important but unglamorous conservation work like long‐term monitoring, resulting in severe information shortfalls (van Rees *et al*., 2022; Maasri *et al*., 2022; see Section IV.4).

Furthermore, funding provided for conservation purposes is often small compared to investments for other societal needs. In the context of enhancing river navigation or flood protection, the alignment of conservation goals with other water‐management goals in a multi‐benefit framework makes strategic use of this disparity (Serra‐Llobet *et al*., 2022). More generally, the integration of conservation opportunities and actions into large‐scale infrastructure projects or other investments may offer opportunities to incentivise action through green financing and integrating nature into decision‐making and operations *via* natural capital (McKay *et al*., 2023; van Rees *et al*., 2023a).

Another major funding gap is to subsidize management decisions that avoid impacts to freshwater biodiversity, for example reductions in fertilizer or pesticide applications or the restoration of riparian buffers in catchment areas with sensitive freshwater ecosystems. Funding schemes like payments for ecosystem services, natural capital investments, and green bonds or green financing might provide support to compensate landowners for foregone capital gains in such circumstances (Park, 2018; Salzman *et al*., 2018). However, the frameworks, metrics, and tools enabling such approaches, and the financial resources to fund them, remain critically underdeveloped (Wiegand *et al*., 2023; Thompson *et al*., 2023).

## V. CUPPING OUR HANDS: USING GAPS TO IDENTIFY SOLUTIONS AND PRIORITIZE INNOVATION

Ultimately, the goal in highlighting gaps and shortfalls is to point the way toward relevant solutions that can bridge important gaps and increase the integrity of global freshwater biodiversity conservation. While a critical review of the many potential strategies for filling the gaps presented herein is beyond the scope of this perspective article, it is illustrative to connect these gaps to an emerging issue in freshwater biodiversity conservation by way of example. Here, we examine key freshwater biodiversity‐related language in the Convention on Biological Diversity's Kunming–Montreal post‐2020 Global Biodiversity Framework (CBD, 2022) through the lens of knowledge, governance, and implementation gaps. Although this exercise is by no means exhaustive, we hope it illustrates how a gap‐based approach can identify the necessary but missing pieces involved with achieving conservation targets and highlight corresponding existing or emerging solutions. A larger list of recommendations, matched with multiple gaps and solutions is presented in Table 4.

**Table 4:** Key language from Target 3 of the Kunming–Montreal Global Biodiversity Framework, associated gaps in knowledge, governance, and implementation, and examples of existing and emerging solutions and recent examples from the freshwater conservation and broader conservation science literature (not necessarily the seminal or earliest publication of the solutions mentioned). Knowledge gaps are highlighted in blue, governance and legislation gaps in red, and implementation and management gaps in green.

Target 3 language	Gaps	Key points	Potential solution(s)	References
‘at least 30% of terrestrial inland and water areas’	Recognition	Severity of ecological decline not recognized by public and policymakers	Citizen science, better or compulsory coverage of conservation topics at all levels of education	De & Dwivedi (2023)
			Communication tools like Safe and Just Boundaries	Rockström *et al*. (2023); Stewart‐Koster *et al*. (2024)
			Public pressure and expectations to influence policymakers	Twardek *et al*. (2021)
			Inclusive and pluralist governance that better integrates non‐Western values of biodiversity	Visseren‐Hamakers *et al*. (2021); Pascual *et al*. (2023)
			Science‐thought leadership	Kaufman & Boxshall (2023)
		Importance of freshwater biodiversity not recognized and appreciated	Storytelling and narrative approaches	Thieme *et al*. (2023)
			Social media and local relationships	Dudo & Besley (2016); Ahmed *et al*. (2022)
			Compilations of evidence, large‐scale syntheses	Lynch *et al*. (2023b)
	Communication	Scientific knowledge not reaching decision‐makers	Clearer conveyance of conservation and management implications	Birnie‐Gauvin *et al*. (2023); Reid *et al*. (2022)
			Communicate risk, build public trust *via* transparency	Paulik *et al*. (2020)
	Funding	Funding for establishing new protected areas insufficient	Other Effective Area‐Based Conservation Measures (OECMs), Nature‐based Solutions (NbS), Natural Infrastructure	van Rees *et al*. (2023b); McKay *et al*. (2023); Watson *et al*. (2023)
		Many current investments contribute to biodiversity loss	Better nature‐positive investments, biodiversity safeguards	Zu Ermgassen *et al*. (2022) Narain *et al*. (2023)
‘Ecologically representative freshwater systems’	Biodiversity inventory	Poor biodiversity knowledge limits understanding of ‘representativeness’	IUCN Global Ecosystem Typology	Keith *et al*. (2022)
		Taxonomic gaps and neglect of cryptic taxa	Novel inventory techniques [e.g. drones, environmental DNA (eDNA)]	Harper *et al*. (2021)
		Geographic bias; underrepresentation of tropics and Global South	Harmonize eDNA practice worldwide; coordinated international funding for underrepresented regions	Belle *et al*. (2019); Moliner Cachazo *et al*. (2023)
		Indigenous People and Local Communities (IPLC) knowledge of species and distributions underrecognized	Ethnobiology approaches, databases of IPLC knowledge	Albuquerque *et al*. (2024)
	Funding	Limited funding for non‐charismatic species and habitats	Freshwater flagship species	Carrizo *et al*. (2017); Kalinkat *et al*. (2017)
			Citizen experts and local knowledge for cost savings	Metcalfe *et al*. (2022); von Gönner *et al*. (2024)
			Focus on freshwater ecosystem services in decision‐maker communication	Lynch *et al*. (2023a)
			Harmonize methodologies for monitoring and management across agencies and partners	Boon *et al*. (2019)
	Planning	Current protected areas may only be representative under present and not future conditions	Explicitly account for anticipated future ranges	Bush *et al*. (2014)
			Permanent *versus* dynamic conservation areas	D'Aloia *et al*. (2019)
			Adaptive management of existing protected areas	Capon *et al*. (2021)
			Prioritize unmodified and unregulated freshwater systems and their catchments as refuges	Capon *et al*. (2021)
			Critical Zone Science approach	Wymore *et al*. (2023)
			Nature Future Framework	Kramer *et al*. (2023)
			Multiple conservation options (historical, hybrid & novel ecosystems)	Erős *et al*. (2023); Lynch *et al*. (2023b)
			Resist–Accept–Direct (RAD) Framework	Ward *et al*. (2023)
‘effectively conserved and managed’	Conservation evidence	Empirical evidence of efficacy of many conservation interventions and management strategies insufficient	Conservation evidence databases, systematic reviews	Acreman *et al*. (2020)
			More rigorous empirical testing of conservation intervention outcomes	Reid *et al*. (2022)
			Measure process in addition to structure in conservation monitoring	Cheung & Burrows (2024)
	Communication	Lack of interdisciplinary collaboration limits effective conservation research and action	Identify key, priority issues for cross‐disciplinary collaboration (e.g. soil conservation, flood control)	Serra‐Llobet *et al*. (2022); McKay *et al*. (2023)
			Ecopracticology	Xiang (2019); Cooke & Birnie‐Gauvin (2022)
			Symposia and working groups	Cooke & Birnie‐Gauvin (2022)
			Raise visibility of non‐English language science, translate scientific terms, provide publication support to non‐native English speakers	Amano *et al*. (2021); Miranda (2023)
		Language barriers impede international collaboration for more effective management	Research distillation into centralized (and multi‐language) national evidence base(s)	Reid *et al*. (2022)
			Artificial intelligence for integrating literature across languages and sources	Berger‐Tal *et al*. (2024)
	Threats and multiple stressors	Current efforts do not address drivers of declines	Strategic portfolios of actions, Social Ecological System framework	Arthington (2021); Birk *et al*. (2020); Thieme *et al. (* 2023); Dudgeon & Strayer (2025)
		Ecological consequences of infrastructure still poorly understood	Integrate ecological modelling with planning processes	Opperman *et al*. (2023); van Rees *et al*. (2022, 2023a)
		New and emerging threats remain underrecognized	Systematic prioritization *via* Delphi method	Aldridge *et al*. (2023)
		Multiple stressors inadequately addressed	Unified and comprehensive Multiple Stressors Assessment Framework	Lima *et al*. (2023)
	Local and bottom‐up	Top‐down, conventional approaches disconnected from local needs	Watershed Health concept	Vollmer *et al*. (2023)
			Integration with local water needs *via* Nature‐based Solutions	van Rees *et al*. (2023b); Vollmer *et al*. (2023)
		Local buy‐in and governance crucial for conservation success	Participatory planning; decision‐making using diverse values	Pascual *et al*. (2023)
	Funding	Conservation funding is often too short‐term or small in scale for effective management	Aligning ecological and societal water needs	van Rees *et al*. (2023b); McKay *et al*. (2023); Erős *et al*. (2023)
			Equitable and sustainable water funds	Bremer *et al*. (2023)
	Scales	Fragmented legislation and governance compromise large‐scale management	Integrative governance	Visseren‐Hamakers *et al*. (2021)
			Enabling government action across scales	Bell‐James (2023)
‘ecosystem functions and services’	Recognition	Links between ecosystem functions and human well‐being not recognized	Storytelling and outreach materials for the public, stakeholder meetings	Thieme *et al*. (2023)
			Social media and stakeholder–scientist relationship‐building	Dudo & Besley (2016); Ahmed *et al*. (2022)
			Alliance for Freshwater Life	Darwall *et al*. (2018)
	Trade‐offs	Ecosystem service delivery and biodiversity conservation goals must be balanced	Economic valuation of biodiversity and ecosystem services, unified indicators for quantification	Loomisa *et al*. (2018); Xu *et al*. (2020)
			Biodiversity accounting and offsetting, credits	Ferreira (2017)
			Ecological Stakeholder Analogs	van Rees *et al*. (2019)
‘Well connected… systems of protected areas’	Scales	Water management differs across scales, complicating connectivity	Meta‐system approach for environmental flows (e‐flows)	Cid *et al*. (2022); Messager *et al*. (2023)
			Watershed‐scale protection and water management measures	Thieme *et al*. (2023)
			Integrative governance	Visseren‐Hamakers *et al*. (2021)
	Natural drivers and ecological processes	Knowledge gaps in geomorphic processes, hydro‐ecological science, hydrological dynamics among habitats	Fluvial biogeomorphology, broader syntheses between biodiversity science and river network dynamics	Doretto *et al*. (2020); Gurnell & Bertoldi (2024)
		Poor understanding of terrestrial, freshwater and marine connectivity	Adaptive management *via* monitoring, decision‐making tools, integration among sustainability objectives	Franklin *et al*. (2024)
			Critical Zone Science approach to river corridor processes	Wymore *et al*. (2023)
‘equitably governed’	Stakeholder engagement	Social equity and environmental justice require careful and ethical engagement with local people and rights‐holders	Ethical co‐production frameworks, convivial conservation	Büscher & Fletcher (2019); Massarella *et al*. (2022)
‘sustainable use’				
		New conservation and sustainability paradigms call for stakeholder co‐production		
‘other effective area‐based conservation measures’				
‘recognizing and respecting the rights of Indigenous peoples and local communities’	Conservation evidence	Evidence base for OECMs, NbS and other shared‐use landscapes or protected areas is lacking	Research and Development monitoring, adaptive monitoring for learning	van Rees *et al*. (2022); Geist & Hawkins (2016)
	Adaptive management	Insufficient monitoring of biodiversity in shared‐used landscapes and watersheds	Participatory modelling for spatial prioritization of monitoring	Flitcroft *et al*. (2023); van Rees *et al*. (2022)
	Communication	Broader interdisciplinary collaboration is a critical missing piece to implementing OECMs and NbS	Symposia, inter‐institutional working groups	van Rees *et al*. (2023b)
		Language barriers and differing epistemologies may impede collaboration between IPLCs and broader scientific community	Artificial Intelligence (AI) translation resources, abstract indexing in different languages	Amano *et al*. (2021); Büscher & Fletcher (2019); Massarella *et al*. (2022)
	Biodiversity inventory	IPLC knowledge of biodiversity, sustainable use and shared landscapes underrecognized	Braided knowledge co‐production, community‐led participatory management	Zhang *et al*. (2020); Brierley *et al*. (2024)
	Trade‐offs	Competing environmental *versus* societal water demand must be managed to protect local communities	Watershed health concept	Vollmer *et al*. (2023)
		OECMs require careful management of water resources trade‐offs	Ecological Stakeholder Analogs	van Rees *et al*. (2019)
	Local and bottom‐up	Conventional conservation approaches may perpetuate disenfranchisement of IPLCs	Indigenous leadership and participation in conservation science	Alexander *et al*. (2021)
			Equity and justice in practice, ‘convivial conservation’	Büscher & Fletcher (2019)

Conserving ‘at least 30% of terrestrial inland and water areas’ (CBD, 2022) will require bridging the Recognition gap to garner public support and get policymakers and decision‐makers across scales to recognize and prioritize the issue. The practice of ‘science–thought’ leadership is intended to facilitate the translation of knowledge across the science‐policy interface; Kaufman & Boxshall (2023) list 11 enabling factors that might facilitate such exchange and increase recognition and action around the freshwater biodiversity crisis. Other authors have suggested myriad strategies for bridging the recognition gap, including increasing public pressure and expectations on policymakers (Twardek *et al*., 2021), providing high‐level communication tools like the Safe and Just Earth System Boundaries (Rockström *et al*., 2023; Stewart‐Koster *et al*., 2024), and distilling large compilations of biodiversity evidence into formats communicable with decision makers (Lynch *et al*., 2023a,b).

OECMs show particular promise as a solution to meeting the funding gap associated with this ambitious Convention on Biological Diversity goal, by including a diversity of restored, anthropogenic, and working waterscapes and thus reducing the need for the acquisition and establishment of new, pristine reserves (Erős *et al*., 2023; Flitcroft *et al*., 2023). NbS and Natural Infrastructure may play an especially large role in addressing funding shortfalls for protected areas by encouraging restoration and protection efforts intended to preserve ecosystem functions for climate adaptation or resilient infrastructure services (McKay *et al*., 2023; van Rees *et al*., 2023a,b). Such integrated management of ecosystems and infrastructure may create opportunities for large‐scale funding through infrastructure agencies, which typically have substantially higher budgets than conservation initiatives. Holistic frameworks for freshwater habitat protection like the Durable Freshwater Protection framework (Higgins *et al*., 2021) may be especially important to ensuring the conservation efficacy of networks of natural, artificial, and mixed‐use hydroscapes in meeting this ambitious area‐based goal.

The Global Biodiversity Framework explicitly references OECMs in conjunction with meeting area‐based conservation goals while promoting ‘sustainable use’ and ‘recognizing and respecting the rights of Indigenous peoples and local communities’ (CBD, 2022), but their broader implementation is hampered by gaps including conservation evidence, communication, and trade‐offs. van Rees *et al*. (2023a) suggest interdisciplinary certificate programs and symposia convening civil engineers, landscape architects, ecologists, and environmental and conservation scientists at national and international scales to bridge the communication gap and promote the interdisciplinary dialogue and collaboration necessary for mainstreaming NbS as OECMs. Conservation evidence approaches like Research and Development monitoring for natural infrastructure (van Rees *et al*., 2022) may be especially valuable in the context of improving and mainstreaming OECMs by facilitating a data‐driven approach to a departure from conventional protected areas. Continued attention to meta‐analytical results of freshwater conservation outcomes (e.g. Acreman *et al*., 2020) and large‐scale evidence syntheses (Reid *et al*., 2022) must be leveraged to vet the potential application of these and other conservation strategies properly.

The goal of specifically conserving ‘ecologically representative freshwater systems’ corresponds directly with the biodiversity inventory gap, and our relatively poor understanding of the diversity and distribution of global freshwater biodiversity. The new IUCN Global Ecosystem Typology for freshwater systems provides a broad‐scale framework with which to approach this issue and prioritize under‐studied habitat types (Keith *et al*., 2022). The interdisciplinary science of ethnobiology, which seeks a greater understanding of human relationships to nature and the epistemologies of environmental knowledge among Indigenous people and local communities may also make substantial contributions towards bridging this gap, as local ecosystems and their resident biodiversity are typically well known to native inhabitants. The combined technologies of environmental DNA (eDNA) and metabarcoding offer increasingly rigorous technological solutions for inventorying even highly biodiverse freshwater communities (Marques *et al*., 2021; Espinosa Prieto *et al*., 2023).

## VI. CONCLUSIONS

(1) The conversations and literature synthesis that led to this perspective revealed that much of the broader freshwater biodiversity conservation literature is focused on highlighting and diagnosing problems, while references to specific solutions are somewhat generalized and limited. The inventory of major knowledge, action, and governance gaps provided in this review is intended to mobilize the global freshwater biodiversity conservation community towards specific solutions for addressing these critical gaps. We highlight 13 cross‐cutting gaps limiting the efficacy and implementation of effective freshwater biodiversity conservation and demonstrate how these can be used to highlight effective solutions in the growing literature around safeguarding freshwater life.
(2) We demonstrate how a gap‐focused analysis can provide actionable, problem‐oriented guidance to achieving objectives from global decision‐makers that are typically phrased using aspirational language. By taking a gap‐focused approach, we can match emerging and existing scholarship to the missing pieces hindering the achievement of these often ambitious objectives, streamlining the identification of context‐specific solutions.
(3) Major knowledge gaps remain surrounding the biodiversity and ecological dynamics of understudied ecosystem types (including intermittent rivers, non‐floodplain wetlands, and ephemeral streams and phreatic systems), their threats, and conservation solutions. Biodiversity in such neglected ecosystems will continue to be lost until these and other critical knowledge gaps are filled. Conservation evidence for freshwater conservation interventions is generally lacking, and support for monitoring will be important to address such gaps.
(4) The multiple overlapping governance structures affecting water resources management and freshwater biodiversity conservation are poorly coordinated across scales and jurisdictional boundaries, creating inefficiencies that impede adaptive and effective conservation. This reduces the adaptive capacity of conservation efforts to change in response to new goals, initiatives, or information, and precludes the necessary large‐scale coordination needed to enact watershed‐scale protections or protect migratory species across river networks or biologically connected wetlands.
(5) Conventional science and policy approaches to sustainable water management and biodiversity conservation remain largely technocratic and top‐down in nature, reducing opportunities for productive dialogue among subject experts, decision‐makers, and local and Indigenous communities. Conventional solutions consequently lack the essential nuance needed for addressing the diverse values of water and the idiosyncrasies of freshwater biodiversity at the local scales at which conservation is implemented.
(6) Strategies for bridging the gap between biodiversity conservation and water management are urgently needed for identifying win–win scenarios in water use, and managing potential trade‐offs if they arise (what we call ‘the trade‐offs gap’). Trade‐offs between human and environmental needs for water are mentioned repeatedly as a research and implementation gap in recent agenda‐setting papers, and will require unprecedented interdisciplinary synthesis and potentially new, context‐specific governance structures to address. Co‐production and participatory exercises with stakeholders, Indigenous communities and other local people are additional critical components to navigating such complexities that are underutilized. Ultimately, the goal of confronting such trade‐offs is to seek mutual gains or win–win outcomes where societal and environmental water needs are met simultaneously. This will require greater integration between water resources management, ecohydrology, and conservation biology to close the gap.
(7) Funding to support freshwater biodiversity conservation continues to be insufficient for achieving meaningful results. Recent, global‐scale enthusiasm for and interest in Nature‐based Solutions (NbS) and natural infrastructure may represent a windfall of multiple‐use funds that contribute to the restoration and conservation of freshwater ecosystems and their resident biota. Given the rapid growth of the literature on freshwater NbS for flood and drought mitigation and stormwater management, growing NbS investments can and should be judiciously leveraged to safeguard freshwater biodiversity.
